# Current practice and preferences to management of equinus in children with ambulatory cerebral palsy: A survey of orthopedic surgeons

**DOI:** 10.1051/sicotj/2019003

**Published:** 2019-02-28

**Authors:** Samuel Gendy, Mohamed ElGebeily, Tamer A. EL-Sobky, Khalid I. Khoshhal, Ayman H. Jawadi

**Affiliations:** 1 Department of Orthopedic Surgery, Hurghada General Hospital Hurghada Egypt; 2 Division of Pediatric Orthopaedics and Limb Reconstruction Surgery, Department of Orthopaedic Surgery, Faculty of Medicine, Ain-Shams University Cairo Egypt; 3 Department of Orthopedics, College of Medicine, Taibah University Almadinah Almunawwarah Saudi Arabia; 4 Department of Pediatric Orthopedic Surgery, King Saud bin Abdulaziz University for Health Sciences Riyadh Saudi Arabia

**Keywords:** Cerebral palsy, Child, Equinus, Pediatric, Physician’s awareness, Professional practice gaps, Silfverskiöld’s test, Surveys and questionnaires

## Abstract

*Introduction*: The consensus among orthopedic surgeons on the management of equinus deformity in cerebral palsy (CP) children has not been reported previously despite being a prevalent deformity. The goals of this study were to examine the orthopedic surgeons’ current practice regarding the management of equinus deformity in children with ambulatory CP, and analyze variations in current practice between general orthopedic and pediatric orthopedic surgeons.

*Methods*: We implemented a brief cross-sectional self-reported questionnaire that addressed the areas of clinical examination and decision-making skills of management of equinus deformity in CP children. We targeted a convenience sample of 400 participants. Surgeons that provided complete responses to the questionnaire were 223 with a response rate of 56%, of which 123 (55%) were general orthopedic surgeons, whereas 100 (45%) were pediatric orthopedic surgeons. The target population consisted of orthopedic surgeons who were further sub-classified in accordance with practice age, general versus pediatric, and exposure to children’s orthopedics during the last three years of their practice. For analytical statistics, the Chi-Square test and Fisher’s exact test were used to examine the relationship between two qualitative variables.

*Results*: The overall clinical practice preferences of all survey participants were unimpressive with discordant survey responses. Pediatric orthopedic surgeons generally demonstrated a statistically significant difference regarding clinical assessment skill items of the survey, in contrast to general orthopedic surgeons. However, we found no differences between pediatric orthopedic and general orthopedic surgeons regarding most of the decision-making/knowledge items.

*Discussion*: Generally, there are insufficient clinical practice trends of both general and pediatric orthopedic surgeons regarding equinus treatment in CP children. This may indicate a knowledge–practice gap with potential risks to CP children undergoing surgery for equinus. There is a need for a more competent exposure to CP in orthopedic surgeons’ educational curricula and an updated health referral system.

## Introduction

**Figure 1:**
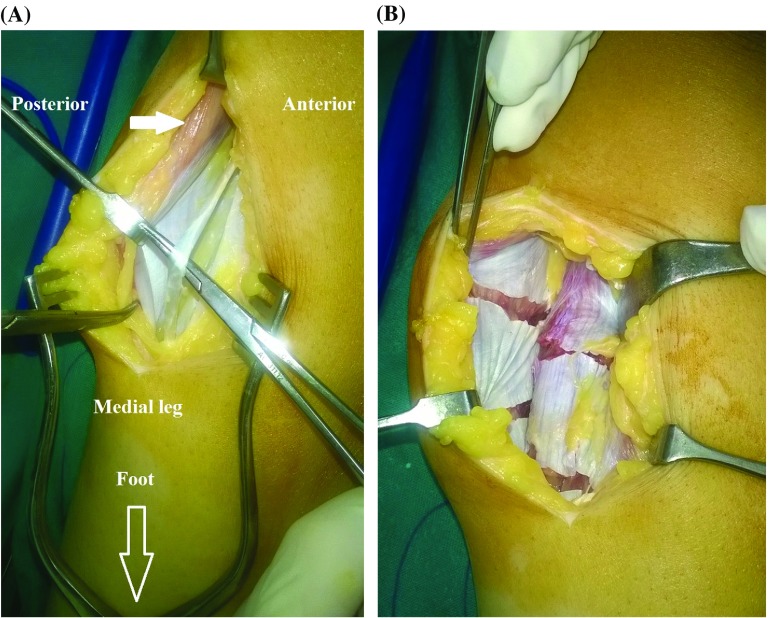
Zone one (proximal) of the triceps surae complex. (A) Note the interface between the ventral aponeurosis of gastrocnemius and dorsal aponeurosis of the soleus with plantaris tendon in between. Part of the gastrocnemius muscle belly is demonstrated (arrow). (B) Aponeurotic lengthening of the gastrocnemius–soleus complex.

Ankle equinus is one of the extremely prevalent deformities in cerebral palsy (CP) children [1]. Equinus contracture is best remedied by lengthening of the triceps surae/gastrocnemius–soleus (gastrocsoleus) complex. Numerous triceps surae lengthening procedures are described in the literature [1,2]. They have been classified in accordance with three distinct anatomic zones of the triceps surae complex. Zone one comprises the medial and lateral bellies of the gastrocnemius, their tendentious insertions, and the soleus muscle belly. Zone two comprises the aponeurotic tendons of the gastrocnemius and soleus, and zone three comprises the Achilles tendon (Figures 1A and B) [2]. Generally, the more proximal procedures are more selective and stable, but are not well suited for severe deformities where greater degrees of correction at the ankle are required. The distal lengthening procedures are less selective and less stable but allow for greater degrees of correction [3,4]. Crouch gait – excessive knee flexion gait – is frequently encountered in ambulatory children with diplegic-bilateral-spastic CP. Loss of plantar-flexion/knee-extension coupling mechanism, muscle weakness, and spasticity are notable risk factors contributing to the development of crouch gait [5,6]. Neglected crouch gait can lead to a significant functional deterioration through the creation of lever-arm dysfunction and eventual joint contractures and bony deformities [5–8]. Contrastingly, imprecise surgical interventions can aggravate the child’s gait inefficiency. This is especially true for the management of ankle equinus. For example, overlengthening, or poorly selected triceps surae lengthening procedure (as an unnecessary Achilles tendon lengthening) can result in the development of a calcaneus deformity, deterioration of crouch gait with diminished ankle push-off moment. This is usually referred to as iatrogenic or “surgeon-induced crouch,” the pathogenesis of which occurs through insulting the soleus an already overburdened muscle in patients with crouch gait [5,6,9]. Likewise, a mistimed – usually early in life – triceps surae lengthening procedure can precipitate deformity [5,6]. Silfverskiöld’s test plays a pivotal role in differentiating gastrocnemius-induced contracture from combined gastrocsoleus-induced contracture. Nonetheless, it is commonly overlooked by physicians [1,6].

Surgeons’ preferences to the management of pediatric CP hip migration [10] and pediatric fractures [11] have been shown to exhibit diversity. Medical surveys are employed to uncover research–practice gaps [10–12]. The consensus among orthopedic surgeons on the management of equinus deformity in CP children has not been reported previously despite being a prevalent deformity. The goal of the present survey study was to examine the orthopedic surgeon’s preferences and current practice to the evaluation and surgical management of equinus deformity in children with ambulatory CP. The specific aim was to identify and analyze variations in current practice and decision-making between general orthopedic and pediatric orthopedic surgeons.

## Materials and methods

### Participant selection

The target population consisted of orthopedic surgeons who were further subcategorized in accordance with practice age, subspecialty – general versus pediatric – and exposure to children’s orthopedic disorders during the last three years of their practice. To achieve this goal, participants were asked to respond to the following three demographic questions: (1) You are practicing orthopedics for? (a) less than five years, (b) more than five years; (2) Are you “permanently” attached to a pediatric orthopedic division at a major institution or identify yourself as having ≥60% of your clinical practice in pediatric orthopedics? (a) Yes, (b) No; (3) Were you exposed to children and adolescents’ disorders during the last three years of your clinical practice? (a) Yes, (b) No. Participants were allocated to the pediatric orthopedic subcategory if they chose “yes” as an answer to question number (2). Otherwise, participants were allocated to the general non-pediatric orthopedic subcategory, irrespective of practice age or exposure to children’s orthopedic disorders during the last three years of their practice. The study was approved by the authors’ institutional review board.

### Survey design

We implemented a brief cross-sectional self-reported questionnaire to enhance response rates, while fulfilling the survey’s objectives. The questionnaire addressed the areas of knowledge and current practice pertaining to history taking, examination, and decision-making skills of the management of equinus in ambulatory CP children (Table 1). According to the survey question type, the questionnaire was subdivided into multiple choice (1–7) and Likert rating-scale questions (8–13) [13]. The clinical assessment of current practice was evaluated by multiple-choice questions (1–5). The decision-making skills and knowledge were evaluated by multiple-choice questions (6, 7) in addition to all rating-scale questions (8–13). The preliminary questionnaire was e-mailed to all co-authors to communicate their feedback. All authors’ concerns were addressed and a final agreement was achieved. To validate the questionnaire, it was revised before use by five senior pediatric orthopedic surgeons other than the authors. The survey was pretested on 20 orthopedic surgeons to explore the accuracy of questions and further enhance the validity of the results. The questionnaire was revised in accordance with the pretest results. In that regard, the second introductory question was revised. The final version was approved and e-mailed to the target population. In the initial invitation e-mail respondents were assured that individual responses would be kept confidential and would never be linked to a respondent’s name. Each individual consented for participation at the beginning of the survey was informed that participation is voluntary, and was given the option to decline. They were also informed of the research topic and that the questionnaire would take approximately 3 min to complete. Reminder e-mails, text messages, and telephone calls were used to enhance response rates. The present study was performed in concordance with reporting guidelines for survey research: an analysis of published guidance and reporting practices [14].

**Table 1:** Questionnaire items and descriptive analysis of all participants: general and pediatric orthopedic surgeons*.

Questionnaire item	Responses	*N*	%
Q1: How often do you take a comprehensive “birth” history from a child with CP?	Regularly/Frequently	177	79
	Occasionally/Never	46	21
Q2: How often do you perform clinical gait assessment for CP children?	Regularly/Frequently	165	74
	Occasionally	47	21
	I am satisfied with a comprehensive couch examination	11	5
Q3: How do you routinely assess your CP child for the presence of equinus contracture?	I dorsiflex the foot above plantigrade, with the hindfoot in neutral and the knee extended.	31	14
	In addition I repeat the above-mentioned maneuver but with a flexed knee “Silverskiold test”.	192	86
Q4: How often do you consult a pediatric neurologist/neurologist when managing CP children?	Regularly/Frequently	142	64
	Occasionally/Never	80	36
Q5: How often do you consult a pediatrician when managing CP children?	Regularly/Frequently	104	47
	Occasionally/Never	119	53
Q6: What is the most common lengthening procedure you use to treat equinus contracture in diplegic CP children?	A percutaneous Achilles tendon lengthening	81	36
	An open Achilles tendon lengthening	78	35
	A more proximal gastrocsoleus aponeurotic lengthening	64	29
Q7: How do you plan for postoperative bracing?	I usually refer patients to rehabilitation specialist to decide on bracing/orthosis	63	28
	I usually instruct my patients to use a specific bracing/orthosis	158	71
Q8: The CP subtype (hemiplegic/diplegic) is an important guide to the selection of the type of lengthening procedure	Totally agree/agree	139	62
	Not sure	62	28
	Totally disagree/disagree	22	10
Q9: The surgeon’s surgical skills is the most important determinant of complication rates (recurrence/overcorrection)	Totally agree/agree	116	52
	Not sure	41	18
	Totally disagree/disagree	66	30
Q10: The choice of the type of lengthening procedure is one of the most important determinants of complication rates (recurrence/overcorrection)	Totally agree/agree	163	73
	Not sure	34	15
	Totally disagree/disagree	26	12
Q11: I do not consider the patient’s age to have a significant influence on complication rates (recurrence/overcorrection)	Totally agree/agree	40	18
	Not sure	31	14
	Totally disagree/disagree	152	68
Q12: The severity of equinus deformity is one of the most important factors that determines the choice of the lengthening procedure	Totally agree/agree	165	75
	Not sure	32	14
	Totally disagree/disagree	24	11
Q13: In case of association of equinus with multiple deformities, it’s important to address all indicated deformities in one anesthetic sitting, “single-stage surgery”	Totally agree/agree	153	69
	Not sure	27	12
	Totally disagree/disagree	43	19

* Participants were asked to check one choice that best corresponds to their answer for each of the 13 questions above. For questions 8–13 participants were asked to indicate their level of agreement or disagreement with these questions/statements: (5) Totally agree (4) Agree (3) Not sure (2) Disagree (1) Totally disagree.

### Data management and analysis

The collected data were revised, coded, tabulated, and introduced using Statistical Package for Social Science (SPSS 20, Armonk, NY). Data analysis was done according to the type of data obtained for each parameter. Descriptive statistics were performed to estimate frequency and percentage of non-numerical data. For analytical statistics, the Chi-Square test was used to examine the relationship between two qualitative variables. Fisher’s exact test: was used to examine the relationship between two qualitative variables when the expected count was less than five in more than 20% of cells. *P*-value: level of significance was set to *P* > 0.05: non-significant (NS), *P* < 0.05: significant (S) and *P* < 0.01: highly significant (HS). We assessed correlations between the general non-pediatric orthopedic surgeons and pediatric orthopedic surgeons’ subcategories and their responses to questionnaire in regard to clinical assessment of current practice and surgical decision-making skills with knowledge. We studied the impact of practice age – time spent in orthopedic practice – on questionnaire responses for all participants and for the previously named participant subcategories.

## Results

Out of 400 targeted surgeons, 223 provided a complete response (response rate 56%). Respondents belonged to Egypt and Saudi Arabia.

### Participant demographics

Of the total number of study participants, 41(18%) and 182 (82%) had practiced orthopedic surgery for less than five and more than five years, respectively. Of these 123 (55%) were general non-pediatric orthopedic surgeons, whereas, 100 (45%) were pediatric orthopedic surgeons. Of the total number of general non-pediatric orthopedic surgeons, 106 (86%) had been exposed to children and adolescents’ disorders during the last three years of their clinical practice.

### Participant responses

The questionnaire items, descriptive analysis, and overall results of all participants are presented (Table 1). We found a statistically significant difference between pediatric orthopedic surgeons and general orthopedic surgeons regarding the clinical assessment skill items of the survey (Table 2). However, we reported no statistically significant differences between pediatric orthopedic and general orthopedic surgeons regarding most of the decision-making/knowledge items (Table 3). Practice age – time spent in orthopedic practice – showed no statistically significant correlations for all questionnaire responses for all participant categories except for questionnaire items 2, 10, and 13 in the summed participants’ category and item 13 for the general subcategory and items 9 and 13 for the pediatric orthopedic subcategory that were statistically significant.

**Table 2:** Clinical assessment skills of participants: general versus pediatric orthopedic surgeons.

Questionnaire item	General orthopedic surgeons		Pediatric orthopedic surgeons		Chi-square test	
	*N*	%	*N*	%	*P*-value	Sig.
Q1: How often do you take a comprehensive “birth” history from a child with CP?						
Regularly/Frequently	84	68	93	93	<0.001	HS
Occasionally/Never	39	32	7	7		
Q2: How often do you perform clinical gait assessment for CP children?						
Regularly/Frequently	78	63	87	87	<0.001	HS
Occasionally/Never	40	32	7	7		
I am satisfied with a comprehensive couch examination	5	4	6	6		
Q3: How do you routinely assess your CP child for the presence of equinus contracture?						
I dorsiflex the foot above plantigrade, with the hindfoot in neutral and the knee extended	23	19	8	8	0.022	S
In addition I repeat the same above-mentioned maneuver but with a flexed knee “Silverskiold test”	100	81	92	92		
Q4: How often do you consult a pediatric neurologist/neurologist when managing CP children?						
Regularly/Frequently	71	58	71	71	0.048	S
Occasionally/Never	51	42	29	29		
Q5: How often do you consult a pediatrician when managing CP children?						
Regularly/Frequently	61	50	43	43	0.326	NS
Occasionally/Never	62	50	57	57		

*N* number, *CP* cerebral palsy, *HS* highly significant, *S* significant, *NS* non-significant.

**Table 3:** Decision-making skills and knowledge of participants: general versus pediatric orthopedic surgeons.

Questionnaire item	General orthopedic surgeon		Pediatric orthopedic surgeon		Chi-square test	
	*N*	%	*N*	%	*P*-value	Sig.
Q6: What is the most common lengthening procedure you use to treat equinus contracture in diplegic CP children?						
A percutaneous Achilles tendon lengthening	41	33	40	40	0.356	NS
An open Achilles tendon lengthening	48	39	30	30		
A more proximal gastrocsoleus aponeurotic lengthening	34	28	30	30		
Q7: How do you plan for postoperative bracing?						
I usually refer patients to rehabilitation specialist to decide on bracing/orthosis	40	33	23	23	0.099	NS
I usually instruct my patients to use a specific bracing/orthosis	81	67	77	77		
Q8: The CP subtype (hemiplegic/diplegic) is an important guide to the selection of the type of lengthening procedure						
Totally agree/agree	74	60	65	65	0.041	S
Not sure	41	33	21	21		
Totally disagree/disagree	8	6	14	14		
Q9: The surgeon’s surgical skills is the most important determinant of complication rates (recurrence/overcorrection)						
Totally agree/agree	60	49	56	56	0.171	NS
Not sure	28	23	13	13		
Totally disagree/disagree	35	28	31	31		
Q10: The choice of the type of lengthening procedure is one of the most important determinants of complication rates (recurrence/overcorrection)						
Totally agree/agree	89	72	74	74	0.345	NS
Not sure	22	18	12	12		
Totally disagree/disagree	12	10	14	14		
Q11: I do not consider the patient’s age to have a significant influence on complication rates (recurrence/overcorrection)						
Totally agree/agree	25	20	15	15	0.374	NS
Not sure	19	15	12	12		
Totally disagree/disagree	79	64	73	73		
Q12: The severity of equinus deformity is one of the most important factors that determines the choice of the lengthening procedure						
Totally agree/agree	95	78	70	70.70	0.001	HS
Not sure	22	18	10	10.10		
Totally disagree/disagree	5	4	19	19.20		
Q13: In case of association of equinus with multiple deformities, it is important to address all indicated deformities in one anesthetic sitting “single-stage surgery”						
Totally agree/agree	72	58	81	81	0.001	HS
Not sure	19	15	8	8		
Totally disagree/disagree	32	26	11	11		

*N* number, *CP* cerebral palsy, *HS* highly significant, *S* significant, *NS* non-significant.

## Discussion

Accurate birth and family history-taking can aid in differentiating between various etiological subtypes of CP and between CP and other neuromuscular disorders presenting in childhood [15]. Generally, the pediatric orthopedic surgeons demonstrated more competent clinical assessment skills than general orthopedic surgeons. The differences were statistically significant. For example, the fact that pediatric orthopedic surgeons were more inclined toward dealing with pediatric neurologists demonstrates that pediatric orthopedic surgeons placed a greater emphasis on the role of multidisciplinary work in the management of CP children.

Dynamic equinus is treated with conservative measures, such as exercises, serial casting, and botulinum toxin type A injections. These measures aim at delaying or preventing the occurrence of fixed/static equinus, thus alleviating the need for surgery, especially in childhood. But surgical treatment could become necessary once fixed equinus develops after failure of the conservative measures [16–18]. Faulty choice of the lengthening procedure may lead to poor clinical outcomes [5,6]. Experimental and clinical studies suggest that gastrocsoleus aponeurotic lengthening procedures can preserve muscle force and other mechanical properties. In that regard, the integrity of the myofascial extensions and the sound healing of the severed aponeurosis are beneficial [19,20]. Contrastingly, the distal Achilles tenotomy is believed to have more deleterious and unanticipated impact on the gastrocsoleus muscle function and biomechanics, especially the push-off strength [1,5,6]. These effects are more pronounced in diplegic/bilateral CP children. Therefore, the recent concepts governing the surgical treatment strategies of fixed equinus in CP children have shifted the emphasis away from Achilles tendon lengthening procedures to the more proximal well-targeted gastrocsoleus aponeurotic lengthening-in-continuity procedures. Likewise, recent trends have shifted the emphasis from early surgery in childhood to delayed surgery in adolescence and from multiple surgical events to single-event multilevel surgery [5–7,9]. Although satisfactory long-term clinical and functional outcomes have been reported with the use of open and percutaneous Achilles tendon lengthening, the above-mentioned recent concepts are strongly supported by kinetic and kinematic and cadaveric biomechanical studies, in addition to clinical and functional findings [6,9,21,22]. Therefore, surgical efforts should be directed toward the more proximal gastrocsoleus aponeurotic lengthening procedures even in severe equinus deformity, where incomplete intraoperative corrections may occur. In such situations, a practical strategy can be splinting the ankle in the under-corrected position and then performing a full correction of equinus after three weeks with or without anesthesia [23]. The fact that experimental studies revealed a delayed onset of muscle-tendon lengthening post-surgical aponeurectomy argues for the above-mentioned clinical practice [20,24]. In contrast to general orthopedic surgeons, statistical analysis demonstrated that pediatric orthopedic surgeons were not simply basing their decisions regarding the choice of the surgical procedure on the severity of equinus.

However, we reported conflicting results regarding the decision-making skills and knowledge of all study participants. This may in part be attributed to the subjectivity of respondents. On the other hand, we reported no statistically significant differences between general and pediatric orthopedic surgeons in regard to the decision-making skills and knowledge, with three notable exceptions that were statistically significant and highly significant, namely questionnaire items 8, 12, and 13. One of these exceptions demonstrated that pediatric orthopedic surgeons were relatively well aware of the importance of differentiating between bilateral and unilateral CP and its relevance to the choice of the surgical lengthening procedure for each subtype. Nevertheless, the fact that most study participants including pediatric orthopedic surgeons tended to adopt the more distal Achilles tendon lengthening procedures on the expense of more proximal gastrocsoleus procedures in diplegic-bilateral CP creates confusion with the above findings. Furthermore, the previous surgical choice and the selection of the Silfverskiöld’s test by all study participants including pediatric orthopedic surgeons as a prime clinical tool to evaluate equinus represents another contradiction. In that regard, it seems that all participants including pediatric orthopedic surgeons were not fully appreciative of the greater importance of preservation of the integrity of the soleus muscle in safeguarding against complications of equinus surgery, namely overcorrection, and subsequent development or worsening of the crouch gait. Hence, the clinical outcomes were not wholly dependent on the physician’s surgical skills.

Triceps surae lengthening procedures are usually performed as part of a single-event multilevel surgery to address lower limb deformities in CP children, which is now the norm among pediatric orthopedic surgeons both in bilateral [5,7,21,25] and unilateral CP children [26]. In contrast to general orthopedic surgeons, statistical analysis demonstrated that pediatric orthopedic surgeons were more mindful of the role of single-event multilevel surgery in the management of equinus in CP children. Both surgeon groups reported unappreciable differences regarding the way they planned for postoperative bracing. This may in part be attributed to logistics and facilities of the work place.

### Knowledge–practice gaps

This study demonstrated knowledge–practice gaps in regard to many aspects of the management of equinus in CP children. The existence of gaps between knowledge and practice has been acknowledged elsewhere in the medical profession including adult orthopedics [27,28]. Knowledge–practice gaps usually manifest in inconsistency in the treatment provided to patients [27]. This can result in suboptimal treatment outcome and overburdening of the health system economy [27,28]. The gap between knowledge and practice has been attributed to various factors, namely constraints of the health system/institution, social and personal factors, cultural beliefs of the medical sector, and the quality of the educational system responsible for delivering the knowledge to practitioners [27].

### Strengths and limitations

A large systematic review analyzed the reporting guidelines for survey research and concluded that there is poor agreement as to the optimal reporting of survey research [14]. The review listed many shortcomings in the quality of medical surveys’ reporting namely, failure to provide access to the study’s questionnaire items and failure to describe the method employed to pretest novel questionnaires among others [14]. In our survey research, we provided a clear description of the questionnaire items, detailed its evolutionary steps, and elaborated on its preliminary validity assessment by a small group of experts and the pretesting by conduction of a pilot study. Yet, we acknowledge that the validity of our questionnaire instrument was not comprehensively evaluated. We also acknowledge that our study population was not exhaustive, but we used a convenience sample that was fairly representative of the target population and described the methods of data analysis and statistics. We did not include non-response analysis nor did we record participants’ age and institutional affiliation. We estimated that such missing variables would be inconsequential to the overall outcomes.

## Conclusion

Generally, there is insufficient clinical practice trends of both general and pediatric orthopedic surgeons with some inconsistencies expressed along various survey elements. Contrastingly, pediatric orthopedic surgeons demonstrated more competent clinical assessment skills. Nevertheless, this competency was not maintainable in regard to the decision-making skills and knowledge. This degree of knowledge–practice gap in both general and pediatric orthopedic surgeons may render CP children with equinus vulnerable to unjustified surgeries and/or higher complication rates such as overcorrection and crouch gait development.

### Recommendations

- Treatment protocols that address management of orthopedic deformities in CP children should be developed, and continuously updated by relevant academic institutions.
- There is a need for a more competent exposure to the management of CP in healthcare professionals’ educational programs.
- A competent and updated health referral system for children with disabilities is on high demand.
- This referral system should ensure appropriate and timely referral of disabled children to their relevant subspecialists at major academic institutions.
- There is a need for continued emphasis on a multidisciplinary approach to tackle childhood disability.
